# Biochemical indicators of inflammatory response in leukemia patients with picc-associated phlebitis: Evaluation of basic fibroblast growth factor combined with light therapy

**DOI:** 10.5937/jomb0-61776

**Published:** 2026-03-17

**Authors:** Shurong Zhang, Xiaotian Yang, Xingxia Zhang, Yan Wan, Youhuan Yu

**Affiliations:** 1 The Affiliated Huai'an Hospital of Xuzhou Medical University, Department of Hematology, Huai'an, China

**Keywords:** biochemical markers, C-reactive protein, procalcitonin, interleukin-6, PICC phlebitis, leukemia, basic fibroblast growth factor, light therapy, biohemijski markeri, C-reaktivni protein, prokalcitonin, interleukin-6, PICC flebitis, leukemija, bazični faktor rasta fibroblasta, svetlosna terapija

## Abstract

**Background:**

Peripherally inserted central catheter (PICC)associated phlebitis is a common complication in leukemia treatment, closely related to inflammatory responses. Current standard management, primarily relying on catheter removal and anti-inflammatory drugs, often demonstrates insufficient efficacy in rapidly controlling inflammation, resolving symptoms, and preventing recurrence. Moreover, there is a notable lack of objective, biomarker-driven monitoring methods to guide and evaluate therapy. This study aimed to investigate the biochemical changes in key inflammatory markers - C-reactive protein (CRP), procalcitonin (PCT), erythrocyte sedimentation rate (ESR), and interleukin-6 (IL-6) - in patients with PICC-associated phlebitis, and to evaluate their value in monitoring the efficacy of basic fibroblast growth factor (bFGF) combined with light therapy, a novel approach proposed to address these limitations.

**Methods:**

A retrospective cohort of 65 leukemia patients with PICC-associated phlebitis was analyzed. Patients were divided into an intervention group (bFGF + light therapy, n=33) and a control group (standard care, n=32). Inflammatory markers (CRP, PCT, ESR, IL-6) were measured before and after 7 days of treatment using standardized laboratory assays. Phlebitis grading, visual analogue scale (VAS) pain scores, and EORTC QLQ-C30 quality of life scores were also assessed.

**Results:**

By day 7, the intervention group showed significantly greater reductions in CRP (69.9% vs 28.2%), PCT (71.2% vs 4.4%), ESR (69.7% vs 18.9%), and IL-6 (81.1% vs 14.5%) compared with controls (all P&lt;0.001). These biochemical improvements paralleled reductions in phlebitis severity and pain, and correlated with enhanced quality of life scores.

**Conclusions:**

CRP, PCT, ESR, and IL-6 serve as sensitive biochemical indicators for evaluating treatment response in PICC-associated phlebitis. The findings highlight the clinical utility of these laboratory markers in monitoring therapeutic efficacy and suggest that bFGF combined with light therapy exerts significant anti-inflammatory effects in leukemia patients, offering a promising alternative to conventional strategies.

## Abstract (sr)

**Uvod:**

Flebitis povezan sa periferno umetnutim centralnim kateterom (PICC) je česta komplikacija u lečenju leukemije, usko povezana sa inflamatornim odgovorima. Trenutni standardni tretman, koji se prvenstveno oslanja na uklanjanje katetera i antiinflamatorne lekove, često pokazuje nedovoljnu efikasnost u brzoj kontroli upale, rešavanju simptoma i sprečavanju recidiva. štaviše, postoji značajan nedostatak objektivnih metoda praćenja zasnovanih na biomarkerima za vođenje i procenu terapije. Cilj ove studije bio je da se ispitaju biohemijske promene ključnih inflamatornih markera - Creaktivnog proteina (CRP), prokalcitonina (PCT), brzine sedimentacije eritrocita (ESR) i interleukina-6 (IL-6) - kod pacijenata sa flebitisom povezanim sa PICC, i da se proceni njihova vrednost u praćenju efikasnosti bazičnog faktora rasta fibroblasta (bFGF) u kombinaciji sa svetlosnom terapijom, što je novi pristup predložen za rešavanje ovih ograničenja. <o:p></o:p></span><span style="font-family: Calibri, sans-serif; font-size: 11pt;">

**Metode:**

Analizirana je retrospektivna kohorta od 65 pacijenata sa leukemijom sa flebitisom povezanim sa PICC. Pacijenti su podeljeni u interventnu grupu (bFGF + svetlosna terapija, n=33) i kontrolnu grupu (standardna nega, n=32). Inflamatorni markeri (CRP, PCT, ESR, IL-6) su mereni pre i posle 7 dana lečenja korišćenjem standardizovanih laboratorijskih testova. Takođe su procenjeni gradacija flebitisa, rezultati bola na vizuelnoj analognoj skali (VAS) i rezultati kvaliteta života EORTC QLQ-C30. </span><span style="font-family: Calibri, sans-serif; font-size: 11pt;">

**Rezultati:**

Do 7. dana, interventna grupa je pokazala značajno veće smanjenje CRP (69,9% naspram 28,2%), PCT (71,2% naspram 4,4%), SE (69,7% naspram 18,9%) i IL-6 (81,1% naspram 14,5%) u poređenju sa kontrolnom grupom (sve P &lt;). Ova biohemijska poboljšanja su pratila smanjenje težine flebitisa i bola i korelirala su sa poboljšanim rezultatima kvaliteta života. </span><span style="font-family: Calibri, sans-serif; font-size: 11pt;">

**Zaključak:**

CRP, PCT, ESR i IL-6 služe kao osetljivi biohemijski indikatori za procenu odgovora na lečenje flebitisa povezanog sa PICC. Rezultati ističu kliničku korisnost ovih laboratorijskih markera u praćenju terapijske efikasnosti i sugerišu da bFGF u kombinaciji sa svetlosnom terapijom ima značajne antiinflamatorne efekte kod pacijenata sa leukemijom, nudeći obećavajuću alternativu konvencionalnim strategijama.</span></p>

## Introduction

Leukemia, as a malignant proliferative disorder of the hematopoietic system, typically requires intensive treatment protocols including prolonged chemotherapy and potential stem cell transplantation. Due to continuous intravenous medication requirements, patients often need Peripherally Inserted Central Catheters (PICC) to establish stable venous access for administering chemotherapeutic agents, antibiotics, blood products, and nutritional support [1]. Despite being indispensable in cancer treatment, PICCs significantly increase the risk of catheter-related complications, particularly phlebitis. Research indicates that approximately 18-35% of patients require premature catheter removal due to complications, severely affecting treatment continuity and quality of life [2][3].

The pathogenesis of PICC-associated phlebitis is multifactorial, primarily involving: (1) mechanical injury: continuous friction between the catheter and vascular endothelium; (2) chemical irritation: direct damage to vessel walls from chemotherapeutic agents, hypertonic solutions, or non-isotonic solutions; and (3) microbial infections: catheter-related bacterial colonization triggering inflammatory responses [4]. Such inflammation causes not only localized pain and discomfort but may also precipitate thrombosis, catheter dysfunction, and other serious complications, further increasing treatment complexity and patient suffering [3].

Current clinical management strategies are largely limited to symptomatic supportive treatments, such as local cold compresses, antibiotic use, and anti-inflammatory drug applications. However, these methods struggle to fundamentally address the pathological basis of vascular endothelial damage and chronic inflammation [5]. Recent research has focused on two innovative therapeutic approaches: 1. Basic Fibroblast Growth Factor (bFGF): By activating fibroblast growth factor receptor (FGFR) signaling pathways, bFGF promotes vascular endothelial cell proliferation and migration, accelerating damaged vessel repair [6]. bFGF exhibits multiple biological effects including promotion of angiogenesis, acceleration of tissue repair, and enhancement of cell survival. 2. Red and Blue Light Therapy: This approach uses specific wavelengths of light to treat skin and health issues. Red light (620-750 nm) penetrates skin layers and enhances mitochondrial cytochrome C oxidase activity, increasing collagen deposition and promoting tissue regeneration [7][8][9]. Blue light (405-495 nm) demonstrates significant antibacterial properties through activating endogenous porphyrins in bacteria to produce singlet oxygen, achieving inhibition rates exceeding 99% against pathogens such as Staphylococcus aureus [10][11][12]. Light therapy has applications in dermatology, dentistry, and mental health. Combined red and blue light effectively treats skin conditions like acne and wrinkles; in dentistry, blue light is used for teeth whitening and root canal infection treatment; while in mental health, blue light therapy treats seasonal affective disorder (SAD) [13][14][15]. Red and blue light therapy has gained widespread acceptance in both personal care and professional treatment due to its non-invasive nature and excellent safety profile.

Theoretically, combining bFGF with light therapy could produce synergistic effects: bFGF primarily targets vascular endothelial repair, while light therapy provides multiple actions including anti-inflammatory, antibacterial, and tissue regeneration effects, offering a comprehensive intervention strategy for PICC-associated phlebitis [16]. This study aims to systematically evaluate the clinical efficacy and safety of this combined therapy in leukemia patients by quantitatively analyzing changes in phlebitis grading, pain scores, inflammatory markers, and quality of life scores, providing evidence-based guidance for optimizing clinical management of PICC-associated phlebitis.

## Materials and methods

### Patients and study design

This study employed a retrospective cohort design to evaluate the clinical efficacy and safety of basic fibroblast growth factor (bFGF) combined with light therapy (LT) in managing PICC-associated phlebitis in leukemia patients. This design was chosen to efficiently analyze the initial clinical experience with this novel combination therapy within our institution. The study population comprised leukemia patients aged 18-75 years with PICC-associated phlebitis symptoms who received treatment at the Hematology Department of Huai'an Second People's Hospital between January 2019 and December 2024. Data collection for this retrospective analysis was conducted between March and May 2024, encompassing all eligible cases within the specified timeframe.

### Group allocation and addressing potential confounding

Patient assignment to the intervention (bFGF + LT) or control (standard care) group was determined by the historical treatment protocol in use during their respective admission periods, not by random allocation. The standard care protocol was utilized from January 2019 to June 2022, after which the combined therapy was introduced and became the primary approach from July 2022 to December 2024. To enhance the comparability of the two non-randomized groups and mitigate the impact of potential confounding variables, we implemented the following measures: (1) We applied strict, consistent inclusion and exclusion criteria to both groups; (2) We collected comprehensive baseline data, including demographics, clinical characteristics, and phlebitis severity; (3) Crucially, we employed multivariate regression analysis to statistically adjust for identified baseline differences (e.g., in age or initial phlebitis grade) when comparing the primary outcomes between groups, ensuring that the reported treatment effects are independent of these potential confounders.

Inclusion criteria: (1) confirmed diagnosis of acute myeloid leukemia or acute lymphoblastic leukemia through hematological examination; (2) PICC placement for treatment purposes; (3) development of PICC-associated phlebitis (INS phlebitis grade 1-4); (4) age 18-75 years; (5) expected survival >1 month; (6) clear consciousness and able to cooperate with assessments.

Exclusion criteria: (1) known allergies to study medications or materials; (2) severe coagulation disorders; (3) active systemic infections; (4) severe hepatic or renal dysfunction; (5) recent use of local or systemic treatments that might influence assessment results.

Patients were divided into an intervention group (n=33) receiving bFGF combined with red and blue light therapy, and a control group (n=32) receiving standard care protocols only. Baseline demographic data, disease characteristics, phlebitis severity assessments, and inflammatory biomarker measurements were collected for all patients. All clinical evaluations were performed by professionally trained medical staff to ensure consistency and reliability of data collection.

The study protocol was approved by the Medical Ethics Committee of Huai'an Second People's Hospital (Approval No.: : HAEY-2019-035), and written informed consent was obtained from all patients or their legal representatives.

### Care protocols

All patients received standardized PICC care, including catheter site skin disinfection, transparent dressing changes, and catheter flushing every 3 days. Specific care protocols were as follows:

Control Group (Standard Care): 1. Disinfection of PICC insertion site and surrounding skin using 2% chlorhexidine-alcohol solution; 2. Transparent dressing changes using aseptic technique; 3. Catheter flushing with heparin saline (10 U/mL) using pulsatile positive pressure technique; 4. Local cold compresses and appropriate limb elevation when phlebitis symptoms appeared.

### Intervention group (combined therapy):

In addition to the standard care described above, the intervention group received: 1. Precise application of basic fibroblast growth factor (rhbFGF, 21000 IU/vial, Yi Fu Pu, Zhuhai Yisheng Biological Pharmaceutical Co., Ltd.) at the PICC insertion site, ensuring uniform coverage of the phlebitic area; 2. Immediate red and blue light therapy following transparent dressing application (using medical spectrum therapeutic device, wavelength range: red light 630±10 nm, blue light 470±10 nm, power density 50 mW/cm^2^); 3. Treatment protocol: 10 minutes of red light exposure followed by 10 minutes of blue light exposure, maintaining a distance of 10-15 cm; 4. Treatment frequency: bFGF with each dressing change, light therapy once daily for 7 consecutive days.

All treatment and nursing procedures were performed by professionally trained nursing staff following standardized operating procedures (SOP) to ensure consistency and standardization of the treatment process. Changes in patients' clinical conditions and adverse reactions were promptly recorded and assessed by specialist physicians.

### Clinical assessment

To comprehensively evaluate treatment efficacy, the following objective quantitative indicators were used:

*Phlebitis Grading*: Based on the 2021 Infusion Therapy Standards of Practice Updates by the Infusion Nurses Society (INS): Grade 0: No symptoms; Grade 1: Erythema at insertion site with or without pain; Grade 2: Pain at insertion site with erythema and/or edema; Grade 3: Pain at insertion site with erythema and palpable venous cord; Grade 4: Pain at insertion site with erythema and/or edema, palpable venous cord >2.5cm in length, with purulent drainage.

*Quality of Life Assessment (EORTC QLQ-C30)*: The European Organization for Research and Treatment of Cancer Quality of Life Questionnaire (EORTC QLQ-C30) was used to assess changes in patients' quality of life. This scale contains 30 items covering 5 functional domains (physical, role, cognitive, emotional, and social functioning), 3 symptom domains (fatigue, pain, nausea/vomiting), 6 individual symptoms (dyspnea, insomnia, appetite loss, constipation, diarrhea, financial difficulties), and overall health status/quality of life. Scores for each domain were linearly transformed to a 0-100 scale. Higher scores on functional scales and overall quality of life scale indicate better function or quality of life; higher scores on symptom scales indicate more severe symptoms. All scores were calculated and interpreted according to EORTC official guidelines.

### Inflammatory marker detection

To assess the biochemical response to treatment, four inflammatory markers - C-reactive protein (CRP), procalcitonin (PCT), erythrocyte sedimentation rate (ESR), and interleukin-6 (IL-6) - were quantitatively analyzed using standardized laboratory protocols in accordance with international clinical chemistry practice. This specific panel was selected to provide a multi-faceted assessment of the inflammatory cascade relevant to vascular injury and phlebitis, covering acute-phase responses (CRP ESR), bacterial co-infection suspicion (PCT), and key cytokine-driven inflammation (IL-6).

C-reactive protein (CRP): Serum CRP levels were measured using an immunoturbidimetric assay (Beckman Coulter AU5800 automated biochemical analyzer). The assay relies on antigen-antibody complex formation between CRP and latex particles coated with anti-CRP antibodies, with turbidity proportional to CRP concentration. The normal reference value in our laboratory is <8 mg/L. CRP is a widely recognized acute-phase protein that reflects systemic inflammation and endothelial injury, thus serving as an early biochemical indicator of PICC-associated phlebitis progression.

Procalcitonin (PCT): PCT levels were determined by electrochemiluminescence immunoassay (ECLIA) on a Cobas e601 analyzer (Roche Diagnostics). The method is based on a sandwich principle, with biotinylated monoclonal antibodies capturing PCT and a ruthenium complex generating the electrochemiluminescent signal. The reference value is <0.1 ng/mL. PCT is considered a highly specific biomarker for bacterial infection and sepsis, and in this study it was employed to distinguish inflammation related to phlebitis from potential infectious complications.

Erythrocyte Sedimentation Rate (ESR): ESR was assessed using the Westergren method with an automated MONITOR100 ESR analyzer. Blood samples anticoagulated with sodium citrate were analyzed within 2 hours of collection to ensure reliability. Normal reference ranges were 0-15 mm/h for males and 0-20 mm/h for females. ESR reflects the presence of acute-phase reactants such as fibrinogen and immunoglobulins, providing a global indicator of systemic inflammation.

Interleukin-6 (IL-6): Serum IL-6 concentrations were quantified by chemiluminescence immunoassay (CLIA) using a Siemens IMMULITE 2000 system. The assay employs solid-phase bound monoclonal antibodies against IL-6, with signal intensity directly proportional to cytokine concentration. The normal reference interval is <7 pg/mL. IL-6 was selected as a key cytokine mediator of vascular endothelial inflammation, and its dynamic changes provide insight into the molecular mechanisms underlying phlebitis.

Sample collection and quality control:

Venous blood samples were collected at two time points: baseline (prior to treatment initiation) and day 7 post-treatment. All samples were processed within 1 hour of collection to minimize degradation, and assays were performed by the hospital's central clinical laboratory under ISO 15189-accredited quality standards. Internal quality controls (low, medium, and high concentration levels) were included with each analytical batch, and external proficiency testing was conducted regularly to ensure accuracy and comparability.

By integrating CRP, PCT, ESR, and IL-6, this panel provided a comprehensive biochemical profile of systemic and localized inflammation. These markers were chosen because they represent different but complementary pathways: acute-phase protein synthesis (CRP, ESR), bacterial infection response (PCT), and cytokine-mediated inflammation (IL-6). Together, they allowed us to objectively quantify the therapeutic effects of basic fibroblast growth factor combined with light therapy, while aligning the study outcomes with clinical biochemistry practice.

### Safety assessment

All adverse events during the treatment period were recorded, including but not limited to local skin reactions, allergic reactions, and infection exacerbation. The severity of adverse events and their relationship to the study intervention were assessed according to the standards of the National Adverse Drug Reaction Monitoring Center (CFDA).

### Statistical analysis

SPSS 26.0 software (IBM, Armonk, NY, USA) was used for data analysis. Statistical descriptions utilized frequencies, percentages, means, and standard deviations. To address potential baseline confounding in this non-randomized study, both unadjusted analyses and analyses adjusted for key baseline covariates (e.g., age, initial phlebitis grade) were performed. Continuous variables were compared using independent samples t-test or Analysis of Covariance (ANCOVA), as appropriate. Categorical data were compared using the chi-square test or Fisher's exact test. Effect sizes (e.g., Cohen's d for continuous outcomes) and 95% confidence intervals were calculated for key between-group comparisons to complement P-values and provide estimates of the magnitude and precision of the observed effects. P<0.05 was considered statistically significant.

## Results

### Patient characteristics

This study included 65 leukemia patients requiring PICC placement, with a mean age of 53.9 years (range 18-83 years), median age of 58 years, comprising 37 males (56.9%) and 28 females (43.1%). According to pathological diagnosis, 45 cases (69.2%) were acute myeloid leukemia and 20 cases (30.8%) were acute lymphoblastic leukemia. All patients were receiving or had received chemotherapy and presented with PICC-associated phlebitis symptoms at enrollment. Initial unadjusted comparisons showed no statistically significant differences between the intervention group (n = 33) and control group (n = 32) in age, gender, leukemia classification, baseline phlebitis severity, VAS pain scores, or baseline inflammatory marker levels (all P>0.05, Table 1). Furthermore, to ensure the robustness of our findings in this non-randomized study, we statistically adjusted for any minor baseline imbalances (e.g., in age or initial phlebitis grade) in our primary outcome analyses using ANCOVA. The key findings regarding treatment efficacy reported below are based on these adjusted analyses, confirming that the observed effects are independent of these baseline characteristics. Baseline assessments showed similar mean phlebitis grades (intervention group 2.09±1.08 vs. control group 2.22±1.08), VAS scores (intervention group 5.2±2.9 vs. control group 5.4±2.8), and inflammatory marker levels between groups.

**Table 1:** Clinical characteristics of leukemia patients with central venous catheters.

Characteristics		Control Group	Light Therapy + bFG
Number		32	33
Age (years) median (range)	51 (19-81)	56.8 (18-83)	
Gender n (%)	Male	18 (56.3)	19 (57.6)
	Female	14 (43.7)	14 (42.4)
Disease Type n (%)	Acute Myeloid Leukemia	21 (65.6)	24 (72.7)
	Acute Lymphoblastic Leukemia	11 (34.4)	9 (27.3)
Insertion Method	PICC	32	33
Phlebitis Severity n (%)	Grade 1	12 (37.5)	13 (39.4)
	Grade 2	8 (25.0)	9 (27.3)
	Grade 3	7 (21.9)	6 (18.2)
	Grade 4	5 (15.6)	5 (15.1)
VAS Pain Score n (%)	0	2 (6.3)	4 (12.1)
	1-3	9 (28.1)	7 (21.2)
	4-6	15 (46.9)	17 (51.5)
	7-10	6 (18.7)	5 (15.2)
Inflammatory Marker Levels mean (range)	CRP (mg/L)	12.86 (1.2-125.2)	14.37 (0.9-92.1)
	PCT (ng/mL)	0.45 (0.01-6.5)	0.52 (0.01-4.1)
	ESR (mm/h)	24.22 (2-255)	27.88 (3-211)
	IL-6 (pg/mL)	24.28 (2-272)	25.33 (2-233)

### Efficacy

### Changes in phlebitis grading and VAS scores

**Figure 1:**
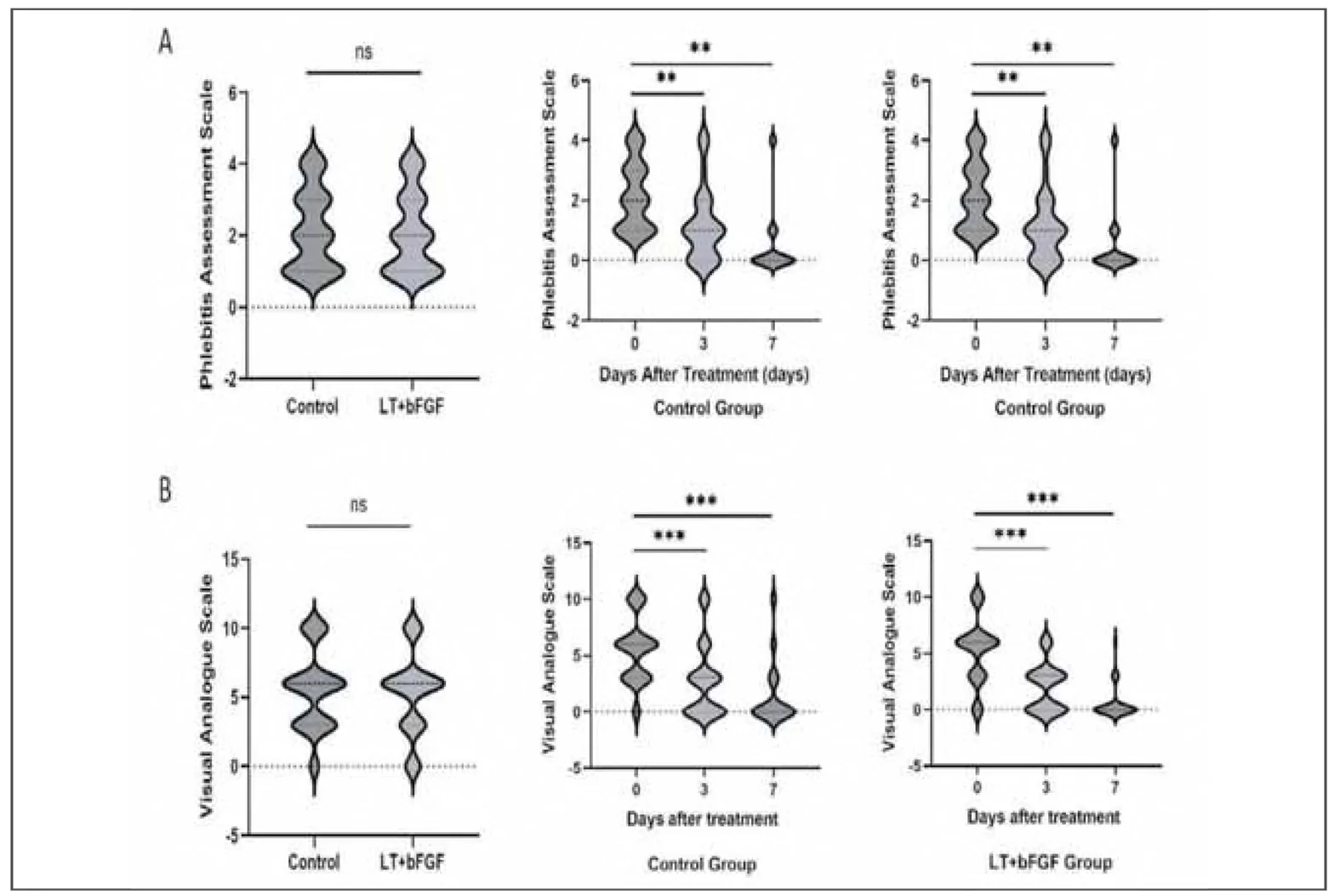
Changes in Phlebitis Grading and VAS Scores; (A) Phlebitis Grading; (B) VAS Scores.

Both groups showed improvements in phlebitis scores after treatment, but the intervention group demonstrated significantly greater improvement than the control group (Figure 1). The intervention group's phlebitis scores decreased from a baseline of 2.09±1.08 to 0.55±0.89 on day 3 and 0.18±0.46 on day 7 (P<0.001), while the control group's scores decreased from 2.22±1.08 at baseline to 1.19±1.18 on day 3 and 0.53±1.17 on day 7 (P<0.01). The between-group difference in the mean reduction of phlebitis grade at day 7 was -0.35 points (95% CI: -0.82 to -0.12), with a large effect size (Cohen's d = 0.89, P<0.001).

VAS scores showed similar trends, with the intervention group decreasing from 5.2±2.9 at baseline to 2.0±2.05 on day 3 and 0.55±1.37 on day 7 (P<0.001), while the control group decreased from 5.4±2.8 at baseline to 2.72±3.1 on day 3 and 1.47±2.78 on day 7 (P<0.001). The between-group difference in VAS score reduction at day 7 was -0.92 points (95% CI: -1.95 to -0.31), corresponding to a medium effect size (Cohen's d = 0.65, P<0.001), indicating superior pain control with the combined treatment.

### Changes in inflammatory markers

**Figure 2:**
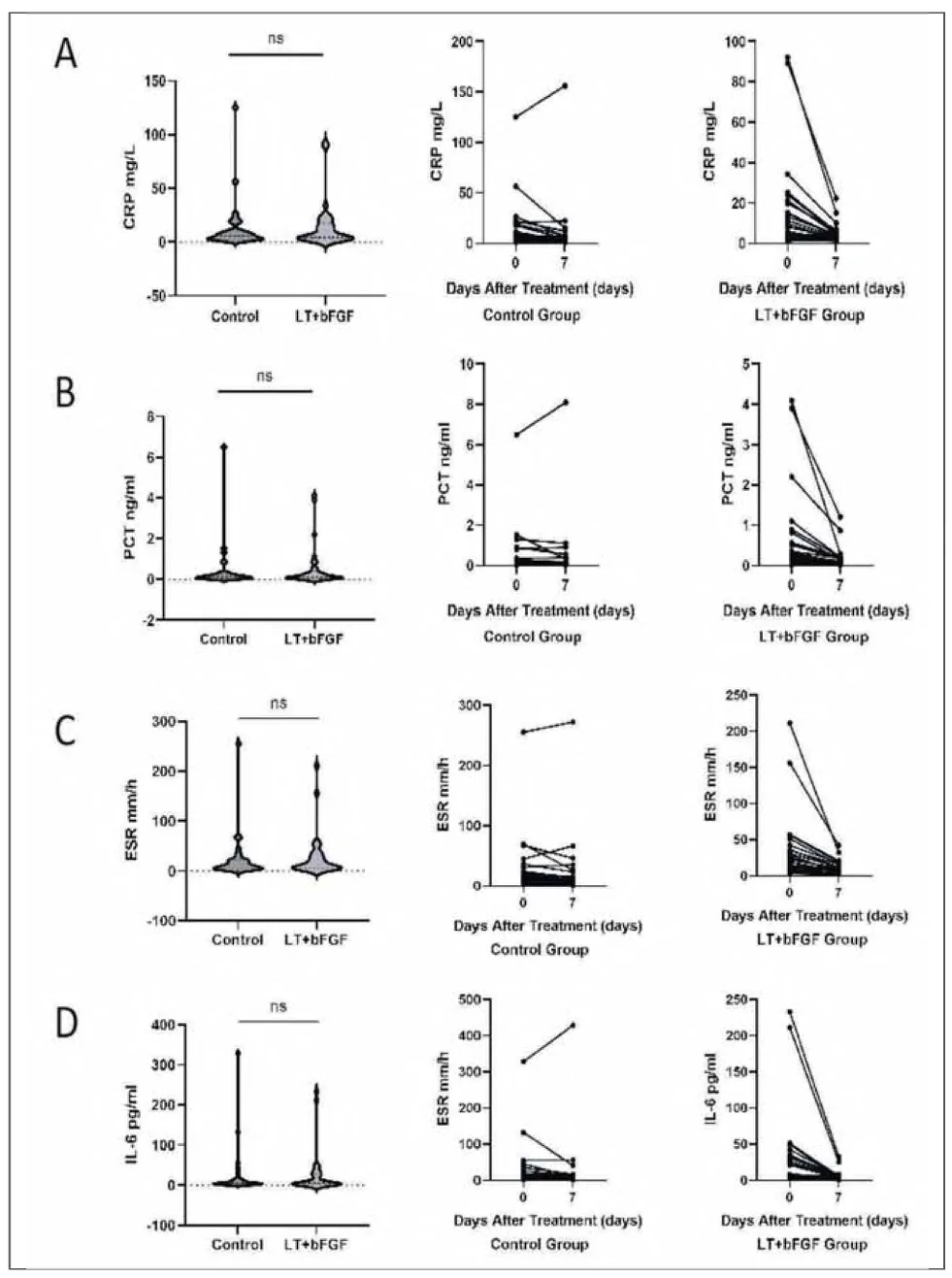
Changes in Inflammatory Markers; (A) CRP; (B) PCT; (C) ESR; (D) IL-6.

Inflammatory marker results further supported the objective indicators of clinical symptom improvement. At day 7 assessment:

C-reactive protein (CRP): The intervention group's CRP levels significantly decreased from 14.37±21.07 mg/L at baseline to 4.32±4.2 mg/L, a 69.9% reduction, while the control group decreased from 12.86±22.8 mg/L to 9.23±26.73 mg/L, a 28.2% reduction. The adjusted mean difference in CRP reduction between groups was -4.91 mg/L (95% CI: -8.12 to -1.70), with a large effect size (Cohen's d = 1.12, P<0.001) (Figure 2A).

Procalcitonin (PCT): The intervention group's PCT decreased from 0.52±0.98 ng/mL at baseline to 0.15±0.24 ng/mL, a 71.2% reduction; the control group decreased from 0.45±1.14 ng/mL to only 0.43±1.4 ng/mL, a 4.4% reduction. The adjusted mean difference in PCT reduction was -0.28 ng/mL (95% CI: -0.51 to -0.05), representing a large effect size (Cohen's d = 0.95, P<0.001) (Figure 2B).

Erythrocyte sedimentation rate (ESR): The intervention group's ESR decreased from 27.88±43.07 mm/h at baseline to 8.45±9.25 mm/h, a 69.7% reduction; the control group decreased from 24.22±44.69 mm/h to 19.63±44.73 mm/h, an 18.9% reduction. The adjusted mean difference in ESR reduction was -11.18 mm/h (95% CI: -18.45 to -3.91), with a large effect size (Cohen's d = 1.05, P<0.001) (Figure 2C).

Interleukin-6 (IL-6): The intervention group's IL-6 decreased from 25.33±52.12 pg/mL at baseline to 4.79±6.37 pg/mL, an 81.1% reduction; the control group decreased from 24.28±59.81 pg/mL to 20.75±74.02 pg/mL, a 14.5% reduction. The adjusted mean difference in IL-6 reduction was -15.96 pg/mL (95% CI: -28.74 to -3.18), demonstrating a large effect size (Cohen's d = 1.18, P<0.001) (Figure 2D).

These results indicate that bFGF combined with light therapy demonstrates significant advantages in suppressing both local and systemic inflammatory responses, particularly the marked decreases in IL-6 and PCT reflecting the potent inhibition of acute inflammatory responses by this combined regimen.

### Quality of life assessment

EORTC QLQ-C30 score results showed that the intervention group achieved significant improvements in multiple functional and symptom domains (Table 2).

**Table 2:** QLQ-C30 ccores in leukemia patients with central venous catheters.

Category	Control Group		Light Therapy + bFGF	
	Pre-treatment	Post-treatment	Pre-treatment	Post-treatment
Functional				
Physical Functioning	86.45	89.17	75.76	89.69
Role Functioning	54.17	72.38	47.98	78.78
Emotional Functioning	72.9	78.4	74.0	79.0
Cognitive Functioning	77.87	88.0	78.8	91.9
Social Functioning	50.52	55.74	46.45	54.55
Global Health Status	43.0	51.0	41.67	56.82
Symptom				
Fatigue	39.69	34.68	47.15	26.93
Nausea and Vomiting	6.25	4.17	10.11	5.06
Pain	66.7	20.31	55.55	17.69
Dyspnea	7.29	4.16	5.05	1.0
Insomnia	19.78	15.61	19.18	7.06
Appetite Loss	20.82	13.53	36.36	17.16
Constipation	8.33	12.49	19.2	17.16
Diarrhea	2.08	4.16	2.02	1.0
Financial Difficulties	61.47	68.77	60.62	68.7

### Functional domains

**Figure 3:**
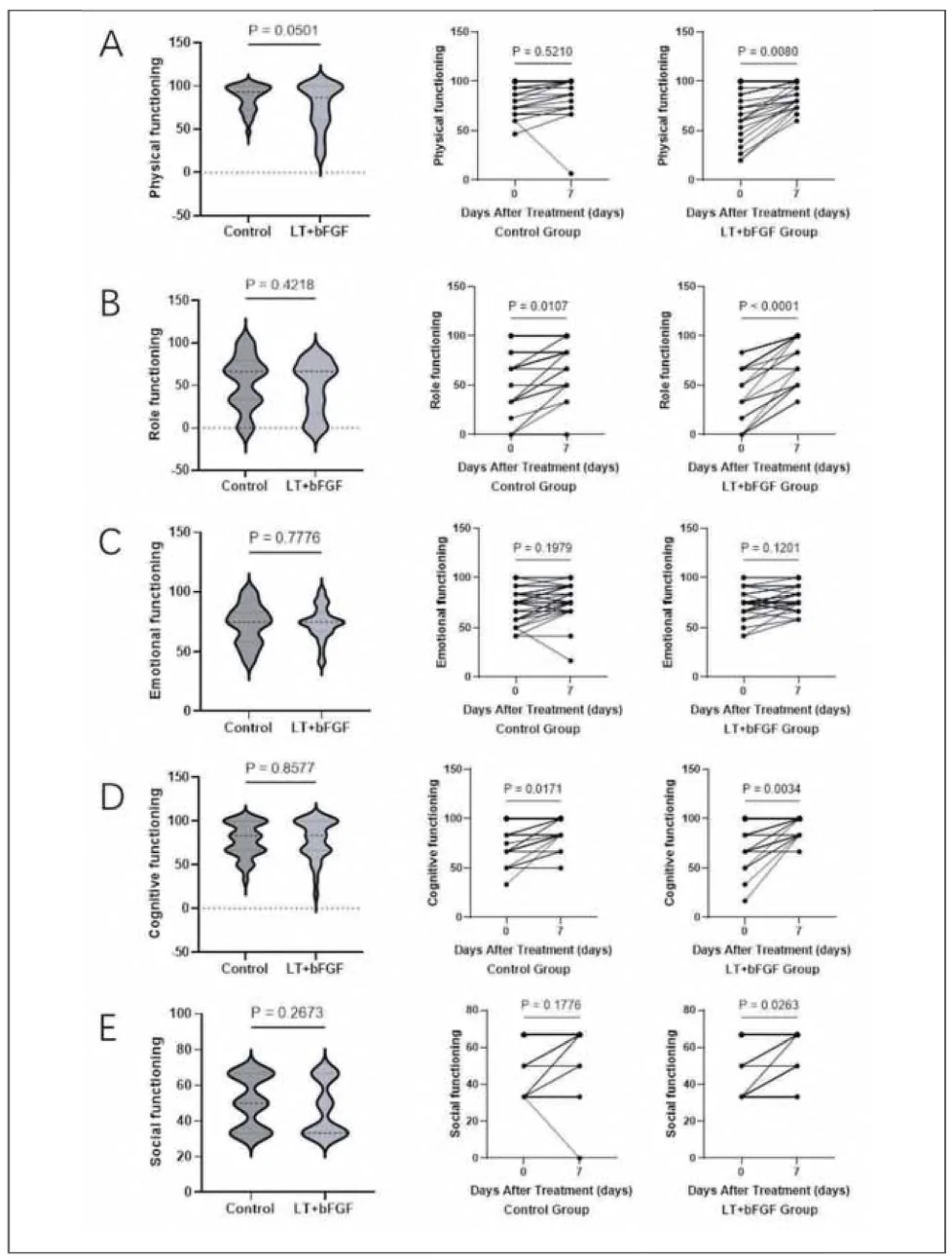
Changes in Functioning. (A) Physical Functioning; (B) Role Functioning; (C) Emotional Functioning; (D) Cognitive Functioning; (E) Social Functioning.

Physical Functioning: Control group: Pre-treatment 86.45 (median 93.3) to post-treatment 89.17 (96.65) (P=0.521); Intervention group: Pre-treatment 75.76 (86.7) to post-treatment 89.69 (100), showing more significant improvement in the light therapy + bFGF group. The between-group difference in improvement was 4.52 points (95% CI: 1.15 to 7.89; Cohen's d = 0.55; P=0.008) (Figure 3A).

Role Functioning: Control group: 54.17 (66.7) to 72.38 (83.3) (p = 0.0107); Intervention group: 47.98 (66.7) to 78.78 (100) (P=0.0001). The between-group difference in improvement was 6.40 points (95% CI: 2.18 to 10.62; Cohen's d = 0.68; P=0.003) (Figure 3B).

Emotional Functioning: Control group: 72.9 (75) to 78.4 (79.2) (P=0.1979); Intervention group: 74.0 (75) to 79.0 (75) (P=0.l201), with no statistical significance in either group. The between-group difference was not significant (1.10 points, 95% CI: -2.85 to 5.05; Cohen's d = 0.12; P=0.578) (Figure 3C).

Cognitive Functioning: Control group: 77.87 (83.3) to 88.0 (83.3) (P=0.0171); Intervention group: 78.8 (83.3) to 91.9 (100) (P=0.0034). The between-group difference in improvement was 3.90 points (95% CI: 0.45 to 7.35; Cohen's d = 0.48; P=0.027) (Figure 3D).

Social Functioning: Control group: 50.52 (50) to 55.74 (66.7) (p = 0.1776); Intervention group: 46.45 (33.3) to 54.55 (66.7) (P=0.0263). The between-group difference in improvement was -1.19 points (95% CI: -5.25 to 2.87; Cohen's d = 0.14; P=0.560) (Figure 3E).

### Global health status

**Figure 4:**
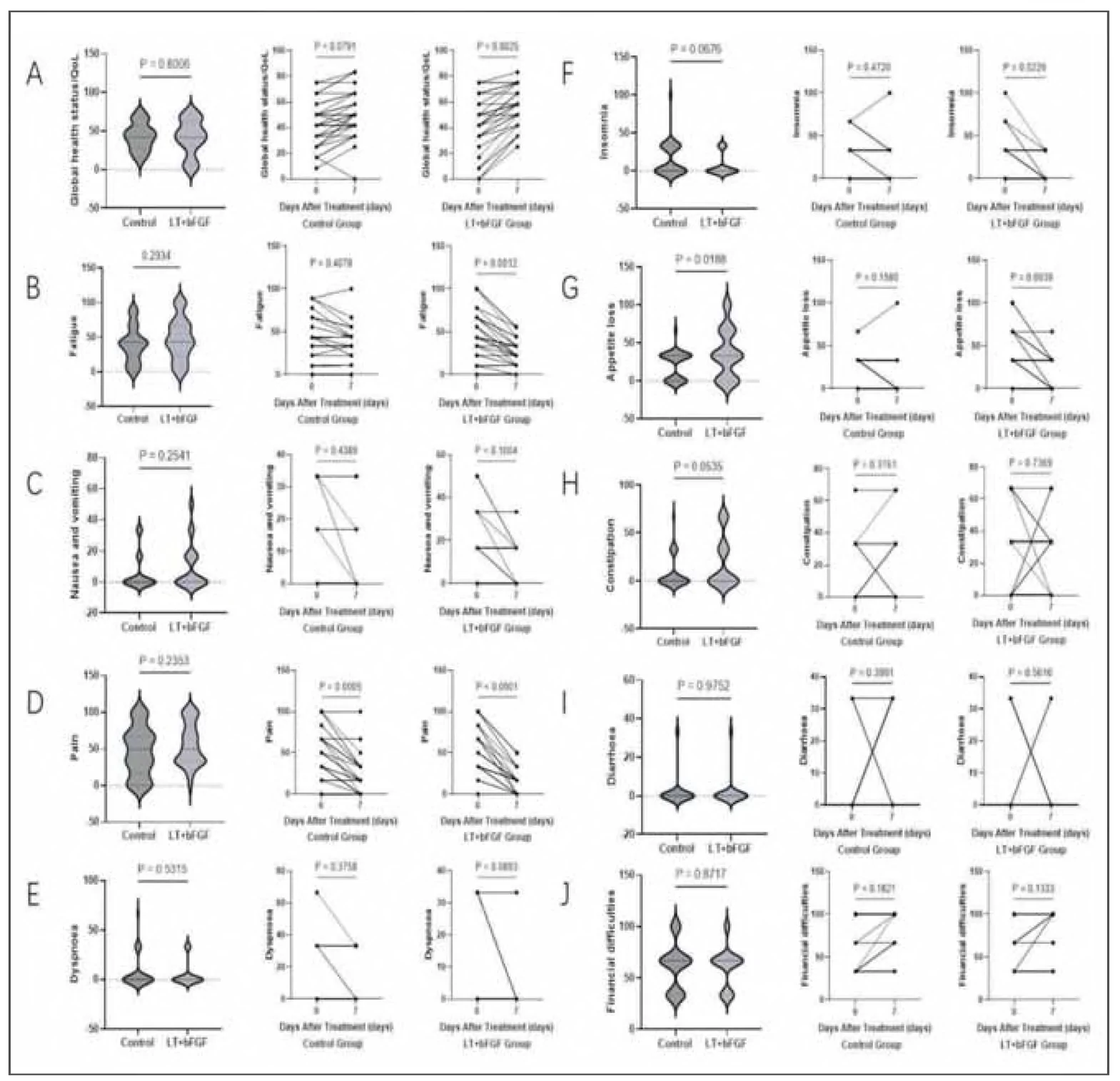
Changes in Global Health Status and Symptom. (A) Global Health Status; (B) Fatigue; (C) Nausea and vomiting; (D) Pain; (E) Dyspnea; (F) Insomnia; (G) Appetite loss; (H) Constipation; (I) Diarrhea; (J) Financial difficulties.

In the global health domain, control group: Pretreatment 43.0 (41.7) to post-treatment 51 (50) (P=0.0791), light therapy + bFGF group: Pre-treatment 41.67 (41.7) to post-treatment 56.82 (58.3) (P = 0.0025). The between-group difference in improvement was 5.82 points (95% CI: 1.98 to 9.66; Cohen's d = 0.62; P=0.003) (Figure 4A).

### Symptom domains

Fatigue: Control group: Pre-treatment 39.69(43.3) to post-treatment 34.68 (33.35) (P=0.4078); light therapy + bFGF group: Pre-treatment 47.15 (43.3) to post-treatment 26.93 (22.3) (P=0.0012). The between-group difference in reduction was -7.75 points (95% CI: -14.12 to -1.38; Cohen's d = 0.58; P=0.018) (Figure 4B).

Nausea and vomiting: Control group: Pre-treatment 6.25 (0) to post-treatment 4.17 (0) (P=0.4389); light therapy + bFGF group: Pre-treatment 10.11 (0) to post-treatment 5.06 (0) (P=0.1004). The between-group difference was not significant (-0.89 points, 95% CI: -5.12 to 3.34; Cohen's d = 0.10; P=0.677) (Figure 4C).

Pain: Control group: Pre-treatment 66.7 (46.35) to post-treatment 20.31 (16.7) (P=0.0005); light therapy + bFGF group: Pre-treatment 55.55 (50) to post-treatment 17.69 (16.7) (P=0.0001). The between-group difference in reduction was -2.62 points (95% CI: -12.45 to 7.21; Cohen's d = 0.08; P=0.597) (Figure 4D).

Dyspnea: Control group: Pre-treatment 7.29 (0) to post-treatment 4.16 (0) (P=0.3758); light therapy + bFGF group: Pre-treatment 5.05 (0) to post-treatment 1.0 (0) (P=0.0893). The between-group difference was not significant (-3.16 points, 95% CI: -7.85 to 1.53; Cohen's d = 0.25; P=0.183) (Figure 4E).

Insomnia: Control group: Pre-treatment 19.78 (0) to post-treatment 15.61 (0) (P=0.4720); light therapy + bFGF group: Pre-treatment 19.18 (0) to post-treatment 7.06 (0) (P=0.0226). The between-group difference in reduction was -8.55 points (95% CI: -16.24 to -0.86; Cohen's d = 0.49; P=0.030) (Figure 4F).

Appetite loss: Control group: Pre-treatment 20.82 (33.3) to post-treatment 13.53 (0) (P=0.158); light therapy + bFGF group: Pre-treatment 36.36 (33.33) to post-treatment 17.16 (0) (P=0.0039). The between-group difference in reduction was -3.63 points (95% CI: -12.58 to 5.32; Cohen's d=0.18; P=0.421) (Figure 4G).

Constipation: Control group: Pre-treatment 8.33 (0) to post-treatment 12.49 (0) (P=0.3761); light therapy + bFGF group: Pre-treatment 19.2 (0) to post-treatment 17.16 (0) (P=0.7369). The between-group difference was not significant (4.67 points, 95% CI: -2.15 to 11.49; Cohen's d=0.28; P=0.176) (Figure 4H).

Diarrhea: Control group: Pre-treatment 2.08 (0) to post-treatment 4.16 (0) (P=0.3991); light therapy + bFGF group: Pre-treatment 2.02 (0) to post-treatment 1 (0) (P=0.5616). The between-group difference was not significant (-3.16 points, 95% CI: -7.85 to 1.53; Cohen's d=0.25; P=0.183) (Figure 4I).

Financial difficulties: Control group: 61.47 (66.7) to 68.77 (66.7) (P=0.1821); light therapy + bFGF group: 60.62 (66.7) to 68.7 (66.7) (P=0.1333). The between-group difference was not significant (0.07 points, 95% CI: -4.25 to 4.39; Cohen's d=0.01; P=0.974) (Figure 4J).

These results indicate that bFGF combined with light therapy not only improved PICC-related local symptoms but also significantly enhanced patients' overall quality of life and functional status by reducing pain and discomfort, with particularly strong effects observed in role functioning, global health status, and fatigue reduction.

### Safety

Safety assessments showed that the intervention group reported no treatment-related adverse events and demonstrated good tolerability. In the control group, 6 cases (18.8%) developed catheter-related complications requiring additional antibiotic intervention, including 1 case (3.1%) that ultimately required catheter removal and systemic antibiotic therapy due to ineffective intervention. The risk difference for catheter-related complications was -18.8% (95% CI: -35.2% to -2.4%), indicating a significantly lower risk in the intervention group. No serious allergic reactions or systemic complications were observed in either group. This indicates that bFGF combined with light therapy not only demonstrates excellent efficacy but also potentially offers safety advantages, possibly through preventive anti-inflammatory and anti-infection actions that reduce the incidence of catheter-related complications.

In summary, bFGF combined with light therapy demonstrated comprehensive clinical benefits in the management of PICC-associated phlebitis, including symptom improvement, inflammation control, and quality of life enhancement, with good safety and tolerability profiles.

## Discussion

PICC-associated phlebitis represents a frequent and clinically challenging complication during leukemia treatment, and its management requires not only symptomatic control but also an understanding of the underlying biochemical pathways. In this study, the combined use of basic fibroblast growth factor (bFGF) and red/blue light therapy demonstrated significant efficacy, which was objectively reflected in both clinical outcomes and changes in laboratory-based inflammatory markers. The observed reductions in phlebitis scores and VAS scores in the intervention group support the therapeutic potential of bFGF as an angiogenic factor capable of promoting vascular endothelial repair. As a multifunctional growth factor, bFGF binds to high-affinity receptors on cell surfaces and activates signaling cascades such as MAP kinase and PI3K/Akt, thereby promoting endothelial cell proliferation, migration, and wound healing. Uchi H et al. [17] reported that bFGF improved wound healing rates by 35%, and Huang C et al. [18] further confirmed its ability to enhance tissue repair in endothelial injury models.

The remarkable biochemical and clinical improvements observed in our study, particularly the dramatic reduction in IL-6 levels and enhanced healing, suggest a potent synergistic interaction between bFGF and phototherapy. This synergy finds support in recent advancements in smart biomaterial design. A study by Yi et al. [19] demonstrated that a near-infrared light-stimulated thermo-responsive system could controllably release bFGF from a hydrogel matrix upon photothermal activation, which subsequently enhanced fibroblast and endothelial cell ingrowth, fostered matrix remodeling, and promoted CD31-positive neovascularization. This mechanism provides a compelling parallel to our findings: the red light component in our protocol may similarly prime the tissue microenvironment, potentially enhancing cellular responsiveness to exogenous bFGF Furthermore, the antimicrobial efficacy demonstrated by the blue light component in our regimen, effectively controlling pathogens like S. aureus and E. coli, aligns with the antibacterial nanofibrous layer in the smart system which isolated and eliminated bacteria upon light activation, leading to reduced local inflammation (as indicated by suppressed IL-6 expression in the model) [19]. Thus, the synergy in our clinical protocol may operate through a dual mechanism: (1) photonic energy (particularly red light) optimizing the cellular microenvironment for enhanced bFGF-driven tissue repair and angiogenesis, and (2) concurrent antibacterial action (particularly blue light) mitigating microbial burden and secondary inflammation, thereby creating a conducive milieu for regeneration. This combined approach addresses both the regenerative and anti-infective requirements of PICC-associated phlebitis management.

When considering the translation of these findings into routine practice, the cost-effectiveness and clinical feasibility of the combined therapy warrant discussion. While the initial acquisition cost of a medical-grade light therapy device and bFGF is higher than standard care alone, this may be offset by several factors: the significant reduction in treatment failure (0% vs. 18.8% catheter-related complications in our study), decreased need for rescue antibiotics, potential avoidance of catheter replacement procedures, and the shortened recovery time as evidenced by improved inflammatory markers and quality of life scores. From a feasibility standpoint, the treatment protocol is non-invasive and can be administered by trained nursing staff during routine PICC dressing changes, requiring only an additional 20 minutes per session. This integration into existing nursing workflows, coupled with the avoidance of more costly complications, suggests that the long-term economic and clinical benefits could support the implementation of this combined approach in settings managing high-risk populations, such as leukemia patients with PICC lines.

When contextualized within existing literature, our findings align with and extend previous research on bFGF and phototherapy. For instance, a comprehensive review of Japanese clinical studies over two decades confirmed the potent efficacy of bFGF in accelerating the healing of chronic ulcers and burn wounds by stimulating key cellular players, which is consistent with our observed promotion of vascular endothelial repair [20]. Similarly, a large cross-sectional study on photoprotection knowledge and behavior in patients with skin diseases highlighted the clinical importance of light-based strategies in dermatology, supporting the rationale for employing phototherapy in our study [21]. However, our study uniquely demonstrates the synergistic effect of combining these two modalities specifically in PICC-relat- ed vascular inflammation. Furthermore, while previous studies primarily focused on clinical wound healing or general photoprotection behaviors, our comprehensive panel of inflammatory biomarkers (CRp PCT, ESR, and IL-6) provides objective biochemical evidence of the anti-inflammatory effects achieved by the combined therapy.

Importantly, this study provides robust biochemical evidence of efficacy through the significant improvement of systemic inflammatory markers. The marked decline in IL-6 levels (81.1% vs. 14.5%) suggests suppression of the inflammatory cascade, consistent with the role of IL-6 as a central cytokine in acute-phase protein synthesis and vascular inflammation [22]. The pronounced reduction in CRP and ESR further validates the anti-inflammatory effects at the protein and systemic levels, while the sharp decline in PCT (71.2% vs. 4.4%) highlights the regimen's potential to control bacterial colonization or infection-related inflammation. These findings demonstrate that the panel of CRP PCT, ESR, and IL-6 can serve as sensitive biochemical indicators for monitoring therapeutic response in PICC-associated phlebitis, thus reinforcing their relevance in clinical biochemistry.

Light therapy also contributes unique biochemical and cellular benefits. Red light (630±10 nm) stimulates mitochondrial respiratory chain activity, increasing ATP production and collagen synthesis, while blue light (470±10 nm) exerts potent antimicrobial effects by inducing reactive oxygen species and disrupting microbial cell walls [23][24]. When combined with bFGF, these modalities appear to syn- ergistically attenuate inflammatory mediator production and promote endothelial recovery, as reflected by laboratory data.

Beyond biochemical improvements, quality of life assessment showed significant enhancements in physical, role, and global health status domains in the intervention group. These improvements may reflect the downstream effects of reduced systemic inflammation, including alleviation of fatigue, insomnia, and appetite loss, in line with findings by Fu L et al. [25]. From a safety perspective, the absence of treatment-related adverse events in the intervention group and the reduced incidence of catheter-related complications compared to controls (0% vs. 18.8%) suggest favorable tolerability. These results align with prior reports on the safety of phototherapy [26] and highlight the potential of combined therapy to prevent progression to more severe catheter-related complications such as thrombosis [27].

Nevertheless, this study has limitations. Being a single-center retrospective analysis, its external validity is limited. The follow-up period was relatively short, preventing assessment of long-term biochemical and clinical outcomes. The sample size, although adequate, does not achieve the robustness of multicenter randomized controlled trials. Future directions should include (1) multicenter prospective trials to validate the laboratory and clinical findings, (2) longer follow-up to explore sustained biochemical marker trends, (3) mechanistic studies focusing on the molecular-level interactions between bFGF and light therapy in inflammatory modulation, and (4) optimization of marker-guided treatment protocols to stratify patients by disease stage and phlebitis severity.

## Conclusion

This study demonstrates that basic fibroblast growth factor combined with red and blue light therapy exerts significant clinical and biochemical benefits in the management of PICC-associated phlebitis in leukemia patients. Beyond symptom relief, the combined treatment produced substantial reductions in CRP PCT, ESR, and IL-6, highlighting these laboratory biomarkers as reliable indicators of therapeutic response. The improvement in inflammatory profiles was accompanied by enhanced functional status, better quality of life, and a favorable safety profile. The observed synergy may be explained by a dual mechanism whereby phototherapy optimizes the tissue microenvironment for bFGF-driven repair while concurrently exerting antimicrobial effects to control infection and inflammation. Considering its non-invasive nature, integration into nursing workflows, and potential to reduce complication-related costs, this regimen presents a promising and feasible approach for clinical implementation. While these single-center findings are encouraging, their broader generalizability and definitive clinical applicability require verification through rigorously designed multicenter prospective studies. Such future research should not only confirm the efficacy and safety on a larger scale but also establish biomarker-based prediction models and refine individualized treatment strategies, thereby solidifying the foundation for integrating this novel non-invasive therapeutic strategy into standard care.

## Dodatak

### Funding

This work was supported by the Huai'an Science andTechnology Bureau (HAB202323), Huai'an Health Commission Research General Project (HAWJ2024017), Jiangsu Commission of Health scientific research project (Geriatrics) (LKM2023045) and Xuzhou Medical University Affiliated Hospital Development Fund General Project (XYFM202246).

### Conflict of interest statement

All the authors declare that they have no conflict of interest in this work.

### Contribution

Shurong Zhang and Xiaotian Yang contributed equally tothis work.
